# Exploring the genetic control of sweat gland characteristics in beef cattle for enhanced heat tolerance

**DOI:** 10.1186/s40104-024-01025-4

**Published:** 2024-05-08

**Authors:** Aakilah S. Hernandez, Gabriel A. Zayas, Eduardo E. Rodriguez, Kaitlyn M. Sarlo Davila, Fahad Rafiq, Andrea N. Nunez, Cristiane Gonçalves Titto, Raluca G. Mateescu

**Affiliations:** 1 Department of Animal Science, North Carolina State University, Raleigh, NC USA; 2 Department of Animal Sciences, University of Florida, Gainesville, FL USA; 3 Ruminant Diseases and Immunology Research Unit, National Animal Disease Centers, United States Department of Agriculture, 1920 Dayton Avenue, Ames, IA 50010 USA; 4 Department of Animal Science, Iowa State, Ames, IA USA; 5 School of Animal Sciences and Food Engineering, University of São Paulo, Av Duque de Caxias Norte 225, Pirassununga, Sao Paulo 13635-900 Brazil

**Keywords:** GWAS, Heritability, Skin, Thermotolerance

## Abstract

**Background:**

Thermal stress in subtropical regions is a major limiting factor in beef cattle production systems with around $369 million being lost annually due to reduced performance. Heat stress causes numerous physiological and behavioral disturbances including reduced feed intake and decreased production levels. Cattle utilize various physiological mechanisms such as sweating to regulate internal heat. Variation in these traits can help identify genetic variants that control sweat gland properties and subsequently allow for genetic selection of cattle with greater thermotolerance.

**Methods:**

This study used 2,401 Brangus cattle from two commercial ranches in Florida. Precise phenotypes that contribute to an animal’s ability to manage heat stress were calculated from skin biopsies and included sweat gland area, sweat gland depth, and sweat gland length. All animals were genotyped with the Bovine GGP F250K, and BLUPF90 software was used to estimate genetic parameters and for Genome Wide Association Study.

**Results:**

Sweat gland phenotypes heritability ranged from 0.17 to 0.42 indicating a moderate amount of the phenotypic variation is due to genetics, allowing producers the ability to select for favorable sweat gland properties. A weighted single-step GWAS using sliding 10 kb windows identified multiple quantitative trait loci (QTLs) explaining a significant amount of genetic variation. QTLs located on BTA7 and BTA12 explained over 1.0% of genetic variance and overlap the *ADGRV1* and *CCDC168* genes*,* respectively. The variants identified in this study are implicated in processes related to immune function and cellular proliferation which could be relevant to heat management. Breed of Origin Alleles (BOA) were predicted using local ancestry in admixed populations (LAMP-LD), allowing for identification of markers’ origin from either Brahman or Angus ancestry. A BOA GWAS was performed to identify regions inherited from particular ancestral breeds that might have a significant impact on sweat gland phenotypes.

**Conclusions:**

The results of the BOA GWAS indicate that both Brahman and Angus alleles contribute positively to sweat gland traits, as evidenced by favorable marker effects observed from both genetic backgrounds. Understanding and utilizing genetic traits that confer better heat tolerance is a proactive approach to managing the impacts of climate change on livestock farming.

## Background

Approximately 45% of beef cattle operations in the United States are stationed in tropical and subtropical locations in the south and southeastern states where hot and humid temperatures are most prevalent [1]. In these environments, cattle compensate for the hotter conditions through eating smaller meals and shifting feed intake to cooler parts of the day [1]. Feed intake has been reported to decline when ambient temperatures reach 25 to 27 °C [2]. In the United States, heat stress decreases productivity leading to an economic loss of $369 million annually [3]. Management strategies such as providing shade, fans, and water, are widely used in the dairy industry to reduce heat stress, but these strategies are costly and difficult to implement in beef cattle operations due to the extensive nature of the production system [4]. To alleviate the effects of hot and humid environments, beef cattle producers use crossbreeding to incorporate a certain proportion of *Bos taurus indicus* genetics in their cattle populations. Brangus is a popular composite breed of 5/8 Angus and 3/8 Brahman used in the southeast regions. The breed combines the superior characteristics of its founder breeds: high meat quality traits from Angus, and adaptability, disease resistance and increased thermotolerance of the Brahman [5, 6]. Thermotolerance can be defined as the ability of an animal to efficiently regulate body temperature in the presence of heat stress, while maintaining a similar level of production. Traits related to sweating competence are important to an animal’s ability to tolerate hot and humid conditions, with heat-adapted cattle capable of increasing sweating rapidly as soon as the skin temperature begins to rise [7]. As air temperatures reach 30 °C, evaporative cooling by sweating is the primary mechanism for heat dissipation in cattle, however, some breeds have a greater potential for heat loss than others [4]. Recent research identified significant differences in skin properties related to heat exchange ability between Brahman and Angus cattle [8]. Studies on the possible genetic control of natural variations in sweat gland properties in beef cattle are not currently available. The objectives of this study were to: 1) characterize sweat gland properties (area, depth, and length) and estimate genetic parameters in a commercial Brangus population, 2) conduct a genome-wide association study (GWAS) on sweat gland properties to identify genetic variants with implications for heat management in beef cattle, and 3) gain insights into the unique genetic architecture of the Brangus breed by conducting a breed of origin GWAS.

## Materials and methods

### Animals and management

The University of Florida Institutional Care and Use Committee approved the research protocol used in this study (Approval No. 201203578). This study utilized 1,681 two-year old commercial Brangus replacement heifers from the Seminole Tribe of Florida, Inc. in Okeechobee FL, and 720 one-year old commercial Brangus replacement heifers from Williamson Cattle Company in Chiefland, FL, USA. Samples and measurements were collected from groups of 150–200 heifers during the summer. This occurred in the following periods: from July 31^st^ to August 21^st^ in 2017, from July 25^th^ to August 15^th^ in 2018, from July 26^th^ to August 9^th^ in 2021, and from July 27^th^ to August 3^rd^ in 2022. Animals from the Seminole Tribe of Florida, Inc. were measured in 8 collection groups during 2017 and 2018, while the Williamson Cattle Company heifers were measured in 4 groups during 2021 and 2022.

### Skin histology preparation

Skin biopsies were collected from the shoulder, 4 inches down from the spine and halfway along the horizontal axis. The skin was cleaned and disinfected using 70% ethanol and 2% chlorhexidine solution (VetOne, Boise, ID, USA), sprayed with 4% Lidocaine Tropical Anesthetic Spray, then punched with a 6-mm biopsy punch (Miltex Inc., York, PA, USA). Biopsies were placed in 10% formalin and stored at room temperature for 16–24 h to allow for fixation. Using a razor blade, biopsies were sliced vertically in half and placed cut side down in 70% ethanol-soaked cassettes. Samples were dehydrated in 70% ethanol, infiltrated in liquid paraffin, and stored until sectioned and stained at the UF Molecular Pathology Core. Sections were cut on a microtome with a thickness of 7 μm and four sections from each animal were placed on one slide and stained with Harris-eosin hematoxylin. Histology slides were photographed using a Nikon T3000 inverted phase microscope (DMZ1200F with NIS Image Elements software) and phenotypes of interest were measured using computer software, ImageJ [9]. One of the four sections was selected based on clear visualization of phenotypes of interest for further analysis. A total area of 1,100 × 1,100 pixels was used on each picture.

### Sweat gland phenotypes

**Fig. 1:**
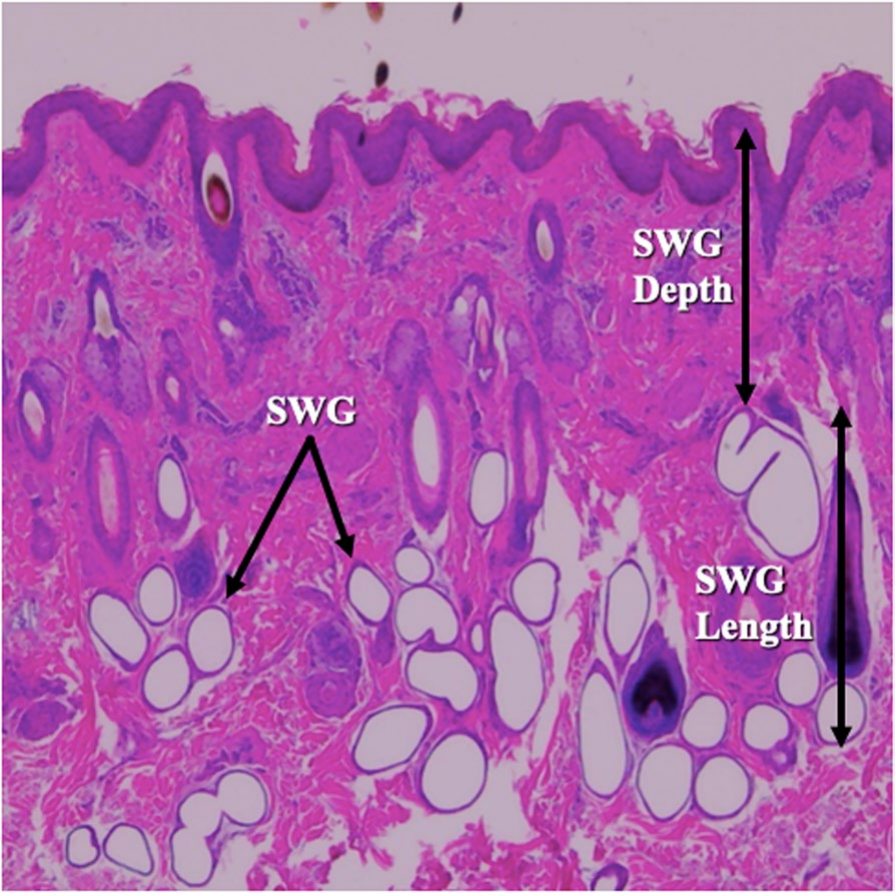
Vertical skin tissue section stained with hematoxyin and eosin. Sweat gland (SWG) area was measured as of the total area ocupied by sweat glands in an 1,100 × 1,100-pixel image. SWG depth was measured as the distance from the top of the sweat gland to the skin surface. SWG length was measured as the distance from the bottom of the sweat gland to the top of the sweat gland

Sweat gland phenotypes included: sweat gland area (μm^2^, Fig. 1) measured as the total area occupied by sweat glands in the 1,100 × 1,100-pixel image section, sweat gland depth (μm, Fig. 1) as the distance from the top of the sweat glands to the skin surface, and sweat gland length (mm, Fig. 1) as the difference between the bottom of the sweat gland to the skin surface and the top of the sweat gland to the skin surface. Sweat gland depth and length were measured in two different locations on each histology slide and the average of the two measurements was used for statistical analysis. Pixels were converted to millimeters using the following conversion formula: 1,000 pixels = 2.145 mm.

### DNA extraction and genotyping

Blood samples were collected from the tail vein and were used to extract DNA, using the QIAamp Mini DNA kit (Qiagen, Valencia, CA, United States). Genotyping was carried out on all animals using the Bovine GGP F250K array (Neogen Corporation, Lincoln, NE, United States) which contains 221,115 single nucleotide polymorphisms (SNPs). SNP positions were mapped using the ARS-UCD 1.2 *Bos taurus* sequence assembly. Using BLUPF90 programs (10), genotypes were filtered for a minor allele frequency of 0.01 and a call rate of 0.90. After quality control, 128,657 SNPs were retained for association analyses.

### Estimation of genetic parameters

Average information restricted maximum likelihood (AIREML) variance components, heritabilities, phenotypic and additive genetic correlations were estimated using single-trait and two-trait genomic best linear unbiased prediction (GBLUP) from single-trait and two-trait animal linear mixed model. Estimates were obtained with airemlf90 package from BLUPF90 family of programs from Ignacy Misztal and collaborators, University of Georgia [10]. The single-trait animal mixed models were as follows:

$$y=\boldsymbol{X}b+\boldsymbol{Z}u+e$$

where *y* is a vector of the observations for the single-trait model; ***X*** is an incidence matrix linking phenotypic records to fixed effects; *b* is a vector of the fixed effects; ***Z*** is an incidence matrix linking phenotypic records to random effects; *u* is a vector of the random animal direct additive genetic effects; and *e* is a vector of the random residual errors for all measured traits and animals. The random effects *u* and *e* were distributed as *u* ~ N(0, $\boldsymbol G\sigma_u^2$) and *e* ~ N(0, $\boldsymbol I\sigma_e^2$), where ${\sigma}_u^2$ is the direct additive genetic variance, ${\sigma}_e^2$ is the residual variance, ***G*** is the genomic relationship matrix, and ***I*** is the identity matrix. The single-trait and two-trait animal mixed models included the collection group (1–12) as a fixed effect. The genomic relationship matrix was based on VanRaden [11], assuming allelic frequency from the population:

$$\boldsymbol{G}=\frac{{\boldsymbol{ZZ}}^{\prime }}{2\Sigma {p}_i\left(1-{p}_i\right)}$$

where ***Z*** is a centered incidence matrix of genotype covariates (0, 1, 2), and the denominator is a scaling parameter, where *p*_*i*_ is the frequency of the reference allele at the *i*-th SNP. The single-trait covariance matrix (V_1_) of *u* and *e* is as follows: $${V}_1=\left[\begin{array}{cc}{\boldsymbol{G}}\sigma_u^{2}& 0\\ {}0& {\boldsymbol{I}}\sigma_e^{{2}}\end{array}\right]$$ 

For the two-trait animal mixed model it was assumed that *u* ~ MVN(0, **T** ⊗ **H**) and *e* ~ MVN(0, **R** ⊗ **I**), where **T** is the additive genetic covariance matrix and **R** is the residual covariance matrix defined between the two traits under analysis, MVN depicts the multivariate normal distribution and ⊗ represents the Kronecker product. The covariance matrix of *u* and *e* random vectors for two-trait model (V_2_) is as follows:

$${V}_2=\left[\begin{array}{cc}\textbf{T}\otimes \textbf{H}& 0\\ {}0& \textbf{R}\otimes \textbf{I}\end{array}\right]$$

### Genome-wide association study

A genome-wide association study (GWAS) was performed for each trait using the weighted single-trait single-step genomic best linear unbiased prediction (WssGBLUP) procedure [12] using 10 kb sliding windows. The airemlf90 package from the BLUPF90 family of programs was used to carry out the computations [10]. The collection group was included as a fixed effect in the single-trait animal mixed models together with the direct additive genetic and residual as random effects.

To estimate SNP effects and weights for this study, the WssGBLUP used an iterative process that was repeated three times. Using this approach, the weights of SNPs with larger effects increase, while those markers with smaller effects decrease. The models used to identify genomic windows associated with sweat gland area, depth, and length included the same fixed and random effects from the variance components. The percentage of direct additive variance explained by a given SNP window was calculated using the formula:

$$\frac{Var\left({w}_i\right)}{\sigma_u^2}\times 100=\frac{Var\left({\Sigma}_{j=1}^B{Z}_j{\hat{a}}_j\right)}{\sigma_u^2}\times 100$$

where *w*_*i*_ is the additive genetic value of the *i*-th kb genomic window, *B* is the total number of adjacent SNPs within the *i*-th window, *Z*_*j*_ is the vector of genotypes of the *j*-th SNP for all individuals, and ${\hat{a}}_j$ is the estimated additive genetic effect for the *j*-th SNP within the *i*-th window. Genomic windows explaining greater than 0.5% of direct additive genetic variance for sweat gland properties were considered to be associated with the analyzed trait and included in subsequent analysis. Manhattan plots were produced using R software [13]. SNPs were mapped to genes using Ensembl version 107 [14] and UCSC ARS-UCD 1.2 genome assembly [15]. The GWAS results are reported as the proportion of genetic variance explained by a 10 kb sliding window.

### Breed of origin GWAS

A GWAS using Breed of Origin Alleles (BOA) was performed to identify regions inherited from particular ancestral breeds that might have a significant impact on sweat gland phenotypes. Quality control was conducted using PLINK. BOA analysis requires stricter quality control and a minor allele frequency of 0.01 and a call rate of 0.99 was applied leaving 93,751 SNPs and 2,261 animals for analyses. BOA were predicted using local ancestry in admixed populations (LAMP-LD), allowing for identification of markers’ origin from either Brahman or Angus ancestry. LAMP-LD utilizes hidden Markov models of haplotype diversity of ancestral populations to trace the origin of alleles in the population [16]. The BOA of the remaining 93,751 SNPs were converted into a pseudo-genotype format, where 0 represented homozygous Angus (AA), 1 represented the heterozygous (AB), and 2 represented homozygous Brahman (BB). These pseudo genotypes were used to conduct a BOA GWAS, utilizing the same methodological approach as was applied to the SNPs.

## Results

### Sweat gland phenotypes

Summary statistics for the sweat gland phenotypes, including the number of animals and the mean and standard deviation, minimum, maximum, and coefficient of variation, are presented in Table 1. The coefficient of variation varied from moderate for sweat gland depth (17.82%) and high for sweat gland area (39.96%).

**Table 1:** Descriptive statistics for sweat gland properties in a Brangus population

Trait	N	Mean	SD	Min	Max	CV, %
Sweat gland area, μm^2^	2,353	287.99	115.08	6.41	966.99	39.96
Sweat gland depth, μm	2,352	936.55	166.86	349.12	1824.9	17.82
Sweat gland length, μm	2,350	624.74	144.91	127.16	1144.2	23.20

### Estimation of genetic parameters

Heritability estimates for sweat gland properties are presented in Table 2. Heritability was estimated to be 0.42 for sweat gland area, 0.28 for sweat gland depth, and 0.17 for sweat gland length. Very few heritability estimates exist for all sweat gland properties. Phenotypic and genetic correlations for sweat gland properties are presented in Table 2. Genetic correlations for sweat gland area were moderately negatively (−0.21) correlated with depth but strongly positively correlated (0.96) with length. Sweat gland depth was weakly correlated (0.11) with length.

**Table 2:** Genetic parameters with standard errors for sweat gland properties in Brangus population

Trait	Sweat gland area	Sweat gland depth	Sweat gland length
Sweat gland area, μm^2^	0.42 ± 0.05	−0.08 ± 0.02	0.53 ± 0.01
Sweat gland depth, μm	0.21 ± 0.10	0.28 ± 0.04	0.03 ± 0.02
Sweat gland length, μm	0.96 ± 0.04	0.11 ± 0.15	0.17 ± 0.04

Heritability estimates are presented along the diagonal, genetic correlations below the diagonal, and phenotypic correlations above the diagonal

### Genome-wide association study

**Fig. 2:**
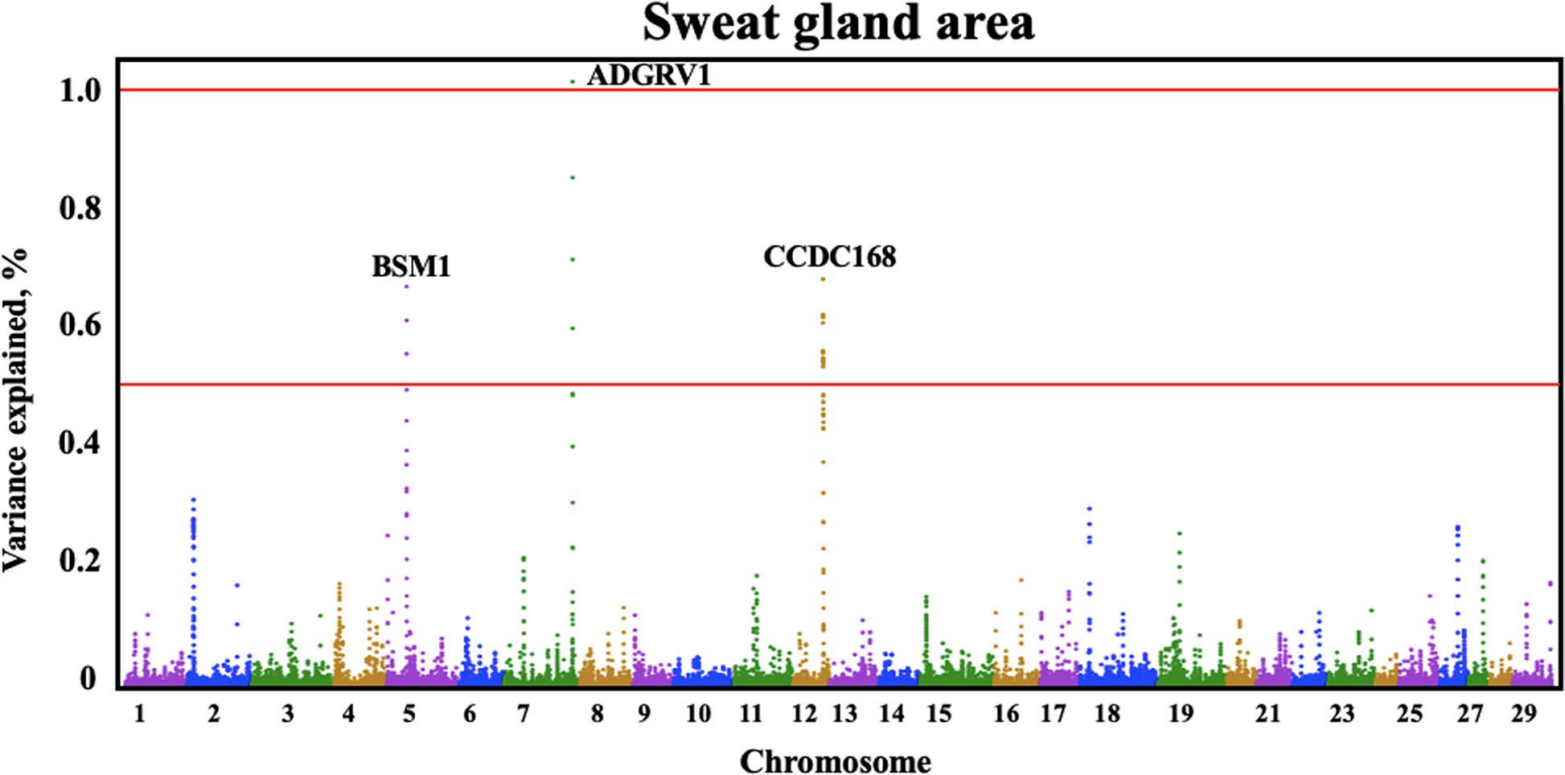
Manhattan plot for genome-wide association analysis for sweat gland area in a Brangus population. Significant thresholds indicated at 1% and 0.5% of the additive genetic variance (solid red line). The variance explained by 10-kb genomic windows was estimated using single trait WssGBLUP analyses

**Fig. 3:**
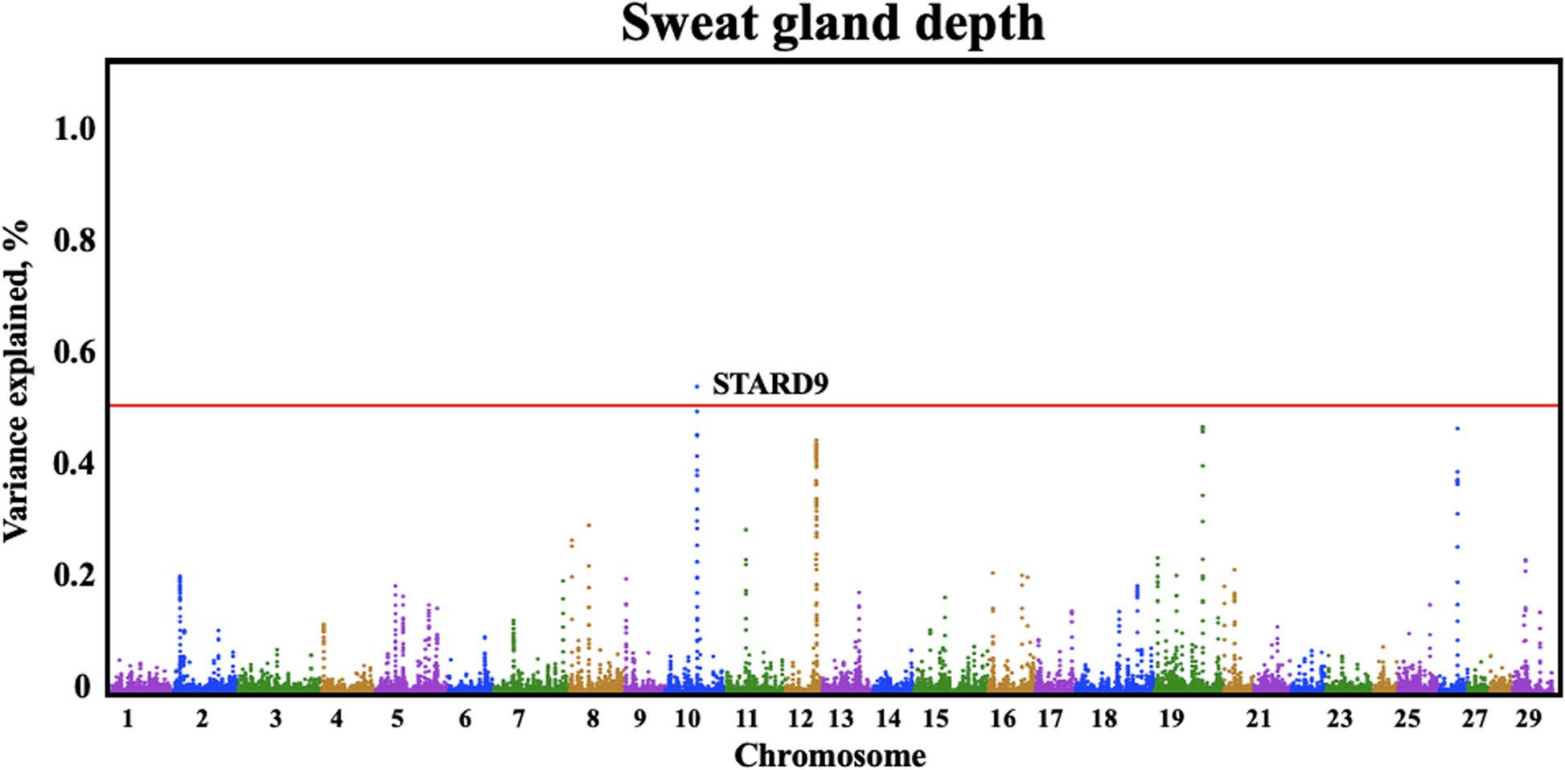
Manhattan plot of bovine chromosomes for sweat gland depth in a Brangus population. Significant thresholds indicated at 1% of the additive genetic variance (solid red line). The variance explained by 10-kb genomic windows was estimated using single trait WssGBLUP analyses

**Fig. 4:**
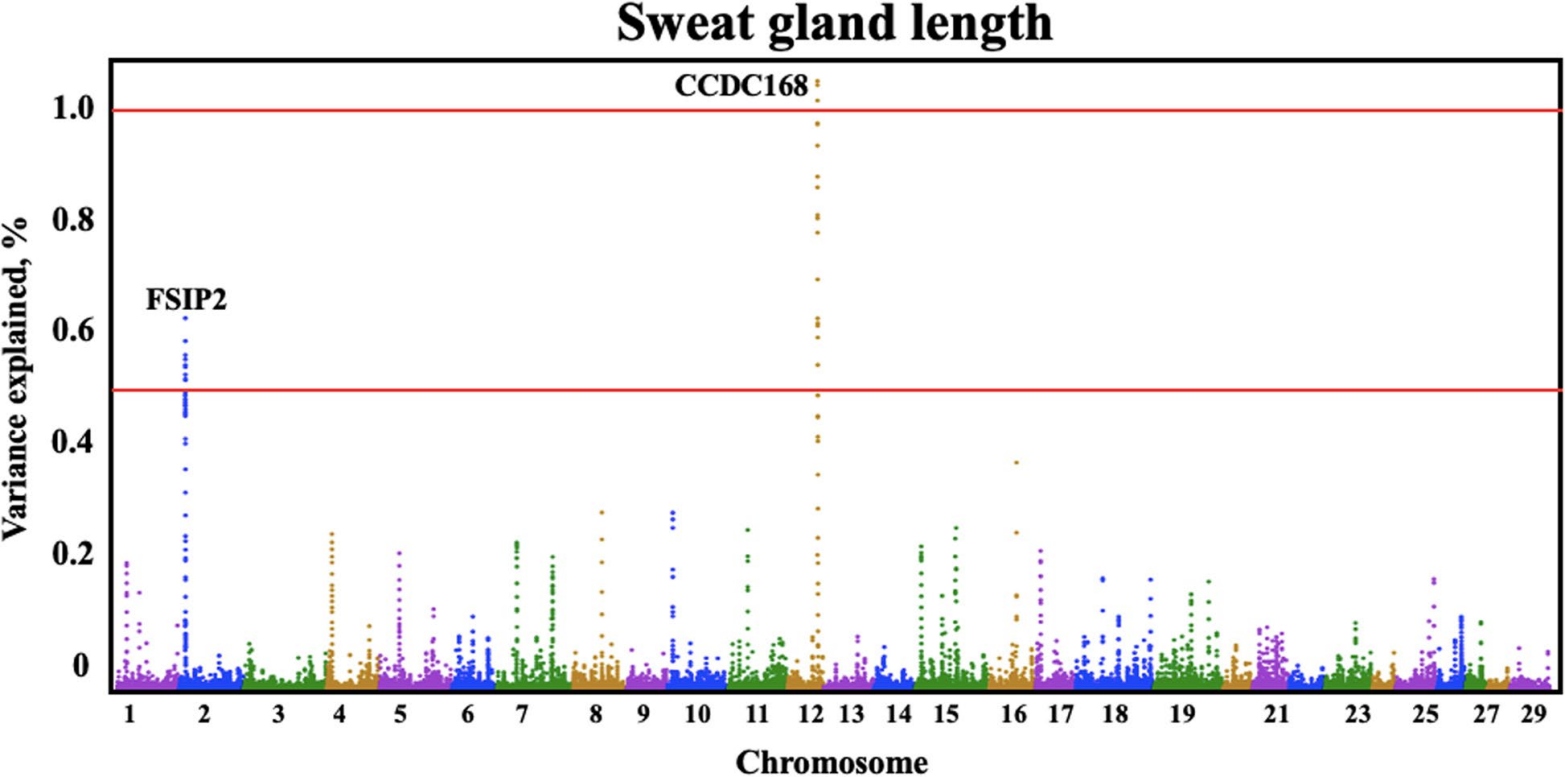
Manhattan plot of bovine chromosomes for sweat gland length in a Brangus population. Significant thresholds indicated at 1% and 0.5% of the additive genetic variance (solid red line). The variance explained by 10-kb genomic windows was estimated using single trait WssGBLUP analyses

Quantitative trait loci (QTLs) were identified by GWAS for all three sweat gland phenotypes (Table 3). Three QTLs were identified for sweat gland area on BTA7, 12, and 5, explaining 1.03%, 0.69%, and 0.68% of the genetic variation, respectively. The BTA7:9,033,0417–90,340,417 genomic window contained one large QTL for sweat gland area (Fig. 2) explaining over 1.03% of the genetic variance. Another large QTL explaining over 0.69% of genetic variance for sweat gland area was located on BTA12. The QTL on BTA5 captured over 0.68% of the genetic variance in sweat gland area. For sweat gland depth and for sweat gland length, there were multiple QTLs identified (Fig. 3 and Fig. 4).

**Table 3:** Predicted functional effect of variants in genes explaining more than 0.50% of genetic variance in Brangus population

Marker name	Gene name	BTA, bp	Trait	Variance explained	Function
rs466246713	*FSIP2*	2:10,594,035–10,604,035	Length	0.65%	Spermatogenic cell-specific protein associated with spermatogenesis [38]
rs109156478	*BSM1*	5:40,358,610–40,368,610	Area	0.68%	Present in mucous secretions of mammalian respiratory, gastrointestinal, and urogenital tracts [26]
rs109160402	*ADGRV1*	7:90,330,417–90,340,417	Area	1.03%	Essential role in the development of hearing and vision [39]
rs210984813	*STARD9*	10:37,986,657–37,996,657	Depth	0.50%	Enables microtubule binding activity [30]
rs208908690	*CCDC168*	12:78,967,164–78,977,164	Area	0.69%	Could have processes related to uncontrolled cell growth [31]
rs208591441	*CCDC168*	12:78,965,942–78,975,942	Length	1.07%	Could have processes related to uncontrolled cell growth [31]

### Breed of origin GWAS

**Fig. 5:**
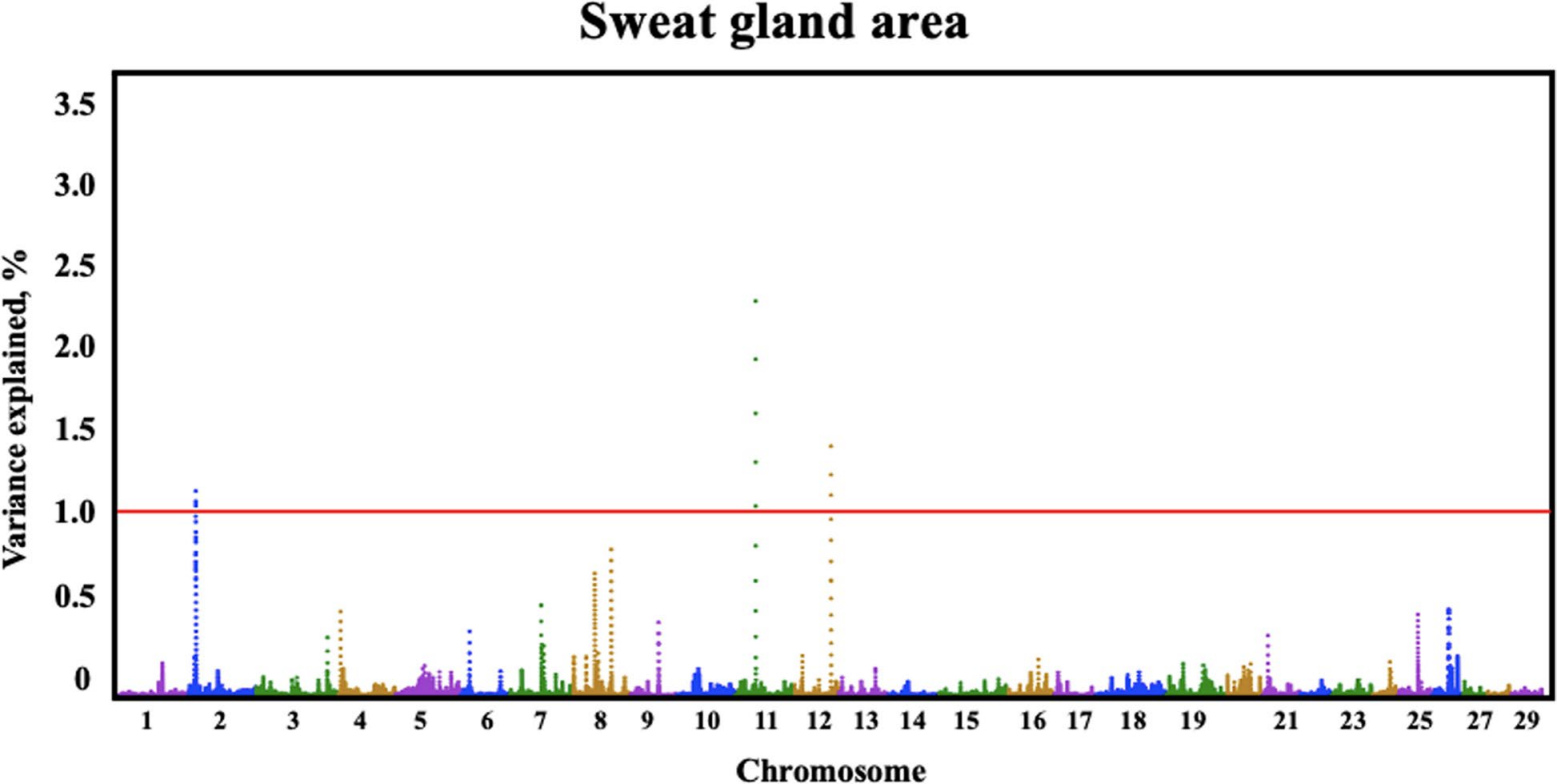
Manhattan plot for breed of origin genome-wide association analysis for sweat gland area in a Brangus population. Significant thresholds indicated at 1% of the additive genetic variance (solid red line). The variance explained by 10-kb genomic windows was estimated using single trait WssGBLUP analyses

**Fig. 6:**
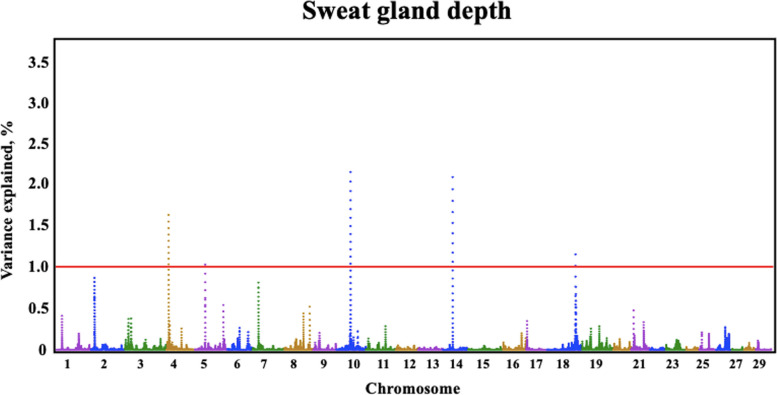
Manhattan plot for breed of origin genome-wide association analysis for sweat gland depth in a Brangus population. Significant thresholds indicated at 1% of the additive genetic variance (solid red line). The variance explained by 10-kb genomic windows was estimated using single trait WssGBLUP analyses

**Fig. 7:**
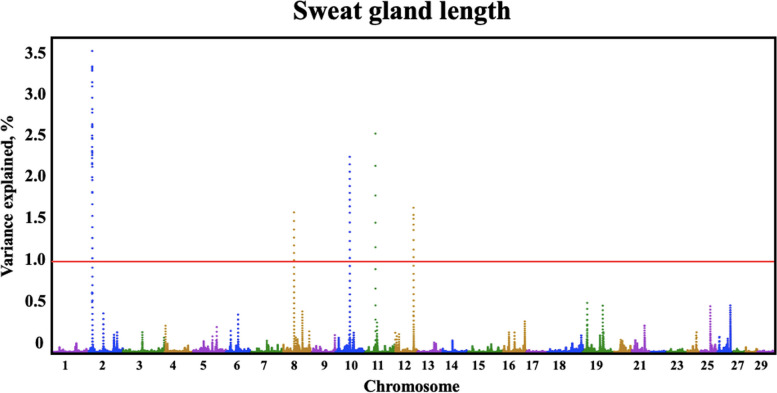
Manhattan plot for breed of origin genome-wide association analysis for sweat gland length in a Brangus population. Significant thresholds indicated at 1% of the additive genetic variance (solid red line). The variance explained by 10-kb genomic windows was estimated using single trait WssGBLUP analyses

Results from BOA GWAS, shown in Fig. 5, 6 and 7, revealed significant QTLs for sweat gland properties. Least square means estimates of the effect on ancestral alleles on each sweat gland property are presented in Table 4.

**Table 4:** Genetic variants explaining more than 1.0% of genetic variance from BOA GWAS in Brangus population

Trait	Marker	BTA, bp	Variance explained	LS means ± SE		
				AA	AB	BB
Sweat gland area, mm^2^						
	rs208728455	2:10,595,942	1.18%	278 ± 3.19^a^	257 ± 14.05^a^	291 ± 3.36^b^
	rs208688454	11:37,770,500	2.28%	289 ± 3.05^a^	243 ± 32.24^ab^	277 ± 3.45^b^
	rs211016058	12:78,967,164	1.44%	282 ± 4.09^a^	288 ± 4.44^a^	281 ± 3.48^a^
Sweat gland depth, mm						
	rs468465385	4:7,520,343	1.57%	940 ± 4.69^a^	924 ± 11.87^a^	932 ± 5.12^a^
	rs210984813	10:37,986,657	2.07%	941 ± 4.10^a^	918 ± 9.22^b^	927 ± 7.05^b^
	rs209432649	14:22,336,058	2.01%	939 ± 4.06^a^	911 ± 29.93^a^	928 ± 6.06^a^
Sweat gland length, mm						
	rs208728455	2:10,595,942	3.58%	612 ± 4.12^a^	599 ± 18.15^ab^	628 ± 4.34^b^
	rs436543135	8:52,760,844	1.66%	615 ± 3.94^a^	618 ± 6.68^a^	628 ± 5.92^a^
	rs210984813	10:37,986,657	2.32%	621 ± 3.65^a^	617 ± 8.21^a^	613 ± 6.28^a^
	rs208688454	11:37,770,500	2.59%	626 ± 3.95^a^	615 ± 41.63^ab^	609 ± 4.46^b^

^a,b^ LS means within rows that do not have a common superscript differ (*P* < 0.05). LS means for the sweat gland measures are presented for the genotypic BOA combinations where A = Angus allele and B = Brahman allele

## Discussion

### Sweat gland phenotypes

In cattle, one of the main routes of dissipating heat in hot environments is through sweating [17]. Sweating dissipates heat from the body through cutaneous evaporative cooling [18]. Cattle only lose about 22% of their latent heat through panting while the rest is lost through moisture from the skin surface when air temperature is greater than 30 °C [4]. The objective of this study was to explore skin properties essential for developing strategies to mitigate heat stress in beef cattle, especially in regions with challenging climatic conditions.

The level of biological variation identified in this study within a relatively uniform groups of cattle suggests a range of genetic potential that, if strategically targeted through selection and management, could lead to significant improvements on these traits for heat stress management. This biological diversity is similar to that found in a population ranging from 100% Angus to 100% Brahman, where a coefficient of variation of 49.77% was found for sweat gland area across five breed groups [8].

### Estimation of genetic parameters

To our knowledge, our study is the first report estimating heritability for sweat gland area and depth. Jenkinson et al. [19] estimated heritability of 0.62 for sweat gland length in *Bos taurus taurus* breeds (Ayrshire and Friesian). The difference in the heritability estimated by Jenkinson et al. [19] and the current study could be due to differences in population size and structure, or measurement. These genetic correlations suggest that it may be possible to indirectly select for cattle with longer sweat gland length and shorter sweat gland depth when selecting for greater sweat gland area. These results suggest that a substantial portion of the observed variation in these traits is attributable to genetic factors. This provides producers with a valuable tool for targeted improvement, allowing them to focus on individuals with favorable sweat gland characteristics.

### Genome-wide association study

Within the BTA7:90,330,417–90,340,417 genomic window containing one large QTL for sweat gland area, a missense variant in the adhesion G protein coupled receptor V1 (*ADGRV1*) gene (SNP rs10916040), causes a G to A base pair change resulting in an amino acid change of valine to isoleucine. Sugier et al. [20] investigated the contribution of *ADGRV1* to the ciliary function. When lungs and nasal passages are exposed to pathogens or toxins, mucus acts as a physical barrier trapping inhaled particles while cilia move the mucus layer and particles to the underlying periciliary layer. In humans, mutations in the *ADGRV1* gene causes Usher syndrome type IIC, a ciliopathy characterized by hearing loss and visual impairment [20]. The nasal ciliated epithelium of patients with Usher syndrome were shown to have lower ciliary beat frequency than healthy subjects. In addition, those with Usher syndrome were reported to have high associations with sinusitis and reduced nasal mucociliary clearance [20]. Sölzer et al. [21] inferred possible SNP × heat stress interaction for claw disorders in Holsteins. GWAS for SNP effects × heat stress interaction found *ADGRV1* as a potential candidate gene for claw disorders in heat stress environments. More importantly, ciliary function plays a significant role in the physiological response to heat stress. Ciliary function contributes to cellular thermoregulation by participating in the sensing and transduction of temperature-related signals [22]. Cilia play a role in mediating signals associated with heat stress, such as those involving heat shock proteins and other molecular pathways that help cells adapt to elevated temperatures [21]. Cilia also function as sensory organelles, detecting changes in the cellular environment [23]. In the context of heat stress, ciliary sensors contribute to the cell’s ability to perceive temperature variations and initiate adaptive responses to mitigate the effects of elevated temperatures.

Another large QTL explaining over 0.69% of genetic variance for sweat gland area was located on BTA12. This region has not been previously annotated in cattle. However, this region shared strong homology (78% identity) with the human *CCDC168* gene (rs208908690). Beck reported *CCDC168* somatic mutations in renal cell carcinomas (kidney cancer) in children [24]. *CCDC168* is a protein-coding gene with poorly understood function but it has been found to be mutated in colorectal cancers in humans [24]. This gene could have processes related to uncontrolled cell growth, but further studies need to be completed to grasp a full understanding of its biological function.

A QTL on BTA5 captured over 0.68% of the genetic variance in sweat gland area and peaks on SNP rs110130339 which is located in the bovine submaxillary mucin (*BSM1*) gene. Bovine submaxillary mucin is a natural gel-forming mucin which comes from bovine submaxillary glands [25]. Mucins represent main components of gel-like secretions or mucus secreted by either mucosae or glands [26]. In humans, mucin is known to play an important role in creating a mucous barrier acting as a protective surface on the epithelia and modulates cell adhesion and immunity through altering its expression [27]. In cattle, one of the essential functions of the mucous gel is protection of tissues against dehydration and is essential for proper functioning of digestive, respiratory, and reproductive systems [28]. We can speculate that in hot and humid conditions, the quality and quantity of mucus could influence how effectively the respiratory tract can function, indirectly affecting an animal’s comfort in high-temperature environments.

For sweat gland depth, there were multiple QTLs identified (Fig. 3). SNP rs210984813, a missense variant located in StAR related lipid transfer domain containing 9 (*STARD9*) gene, captured over 0.50% of the genetic variance. This is a missense mutation from G to A/T, causing an amino acid change from serine to asparagine/isoleucine. Moore et al. [29] investigated differentially expressed genes in the endometrium and corpus luteum (CL) between Holstein cows with either good or poor fertility. Heifers with a lower expression of *STARD9* featured compromised CL development and reduced steroidogenic capacity resulting in their genetic merit for calving intervals being in the bottom 5% [29]. Because *STARD9* is known to be associated with specific cellular processes, lipid metabolism, or other functions, it could indirectly contribute to the cellular response to stress, including heat stress. Genes involved in lipid transport or metabolism, such as *STARD9*, could indirectly influence cell membrane properties [30]. Maintaining the integrity and fluidity of membranes is crucial for cellular function under stress conditions. Moreover, heat stress triggers various cellular stress responses, including the activation of specific signaling pathways. Proteins with lipid transfer domains may play a role in these pathways, potentially influencing cellular adaptation and survival mechanisms.

Several QTLs were identified for sweat gland length (Fig. 4). On BTA12, a large QTL explaining over 1.0% of genetic variance was located in coiled-coil domain containing 168 (*CCDC168*) gene. Coiled-coil domains are structural motifs found in proteins, characterized by a helical structure. These domains often facilitate protein-protein interactions and are involved in various cellular processes, including signaling, transport, and structural functions [31]. The specific functions of *CCDC168* may depend on the cellular context, the tissues in which it is expressed, and its interacting partners. Huang et al. [32] explored gene expression patterns of the pituitary gland and hypothalamus of Angus cattle in different developmental and growth stages to identify genes that affect bovine reproductive performance. *CCDC168* was found to be one of the major differentially expressed genes in the pituitary gland that was being upregulated during the growth stage from 6-month to 30-month old Angus [32]. During heat stress, cattle experience decreased reproductive rates, this gene could influence reproductive rates during heat stress events. Furthermore, BTA2 contains a QTL explaining over 0.65% of the genetic variance. Within this region, SNP rs466246713, a missense variant in the fibrous sheath interacting protein 2 (*FSIP2*) gene, causes an A to G base pair change resulting in an amino acid change of histidine to arginine. *FSIP2* is a spermatogenic cell-specific protein associated with spermatogenesis [33]. Zhang et al. [34] found that mutations in *FSIP2* affect the development and progression of testicular germ cell tumors and investigated the relationship between *FSIP2* and renal cell carcinoma (ccRCC) in humans. It was found that *FSIP2* may serve as a potential predictive biomarker for prognosis of ccRCC as it may play a role in metastasis and tumor invasion [34]. Piersanti et al. [35] investigated the transcriptome of granulosa cells during follicle development to determine potential causes of metritis. RNAseq revealed one of the most upregulated genes in granulosa cells during follicular development to be *FSIP2*. Pathway analysis of the differentially expressed genes indicated involvement in immune functions, cell to cell communication, cellular metabolism, and cell cycle [35]. Heat stress often induces the expression of heat shock proteins and molecular chaperones. These proteins help in stabilizing and refolding damaged proteins that may occur under heat stress conditions. If *FSIP2* is involved in protein interactions or structures within cells, it might indirectly be affected by or contribute to the cellular response to heat stress.

### Breed of origin GWAS

Breed of origin GWAS can improve the power to detect genetic variants associated with traits, especially when analyzing populations with multiple ancestral backgrounds. Analyzing the ancestral origins of genomic segments can aid in localizing and pinpointing causal variants within specific ancestral backgrounds. This helps in understanding the functional and evolutionary significance of these variants.

Three SNPs with significant effect on sweat gland area were identified by BOA GWAS. SNP rs208728455, on BTA2, showed a significant increase in sweat gland area when inherited as homozygous Brahman alleles (291 μm^2^) compared to homozygous Angus alleles (278 μm^2^). Conversely, on BTA 11, SNP rs208688454 shows an opposite trend with homozygous Angus alleles having superior sweat gland area (289 μm^2^) compared to homozygous Brahman alleles (277 μm^2^). Marker rs211016058 featured no significant differences in sweat gland area when inherited from Brahman or Angus alleles. Nay et al. [36] found that Zebu sweat glands were larger, longer, and closer to the skin surface, suggesting that these characteristics confer Zebu cattle have a greater potential for moisture loss through sweating than European cattle. Our phenotypes align with these findings, but the BOA GWAS also indicate that genetic variants for increased sweating ability could exist in Angus cattle that are not present or not as pronounced in Brahman cattle.

Three additional SNPs with significant effect on sweat gland depth were identified by BOA GWAS. SNP rs210984813 shows a significant decrease in sweat gland depth when an individual inherits homozygous Brahman alleles (927 μm) compared to homozygous Angus alleles (941 μm). Individuals inheriting heterozygote alleles (918 μm) also featured a significant decrease in sweat gland depth compared to those with homozygous Angus alleles. Our findings align with studies conducted in other animals, Windsnyers pigs were shown to have sweat glands that were more superficial and significantly different from other breeds [37]. While more sweat gland studies need to be undertaken, it’s plausible that this breed has better heat tolerance compared to other breeds due to these sweat gland properties. According to Nay et al. [36], sweating efficiency in cattle appeared to be impacted by the depth of sweat glands. While functionality of the sweat glands continues to be explored, this trait could have an effect on the rate of heat transfer from an animal to the environment.

BOA GWAS identified four SNPs with significant effect on sweat gland length: rs208728455, rs436543135, rs210984813, and rs208688454. SNP rs208728455 and rs208688454 were also significantly associated with sweat gland area while rs210984813 was significantly associated with sweat gland depth. Marker rs208728455 showed an increase in sweat gland length when inherited as homozygous Brahman alleles (628 μm) compared to homozygous Angus alleles (612 μm). Conversely, SNP rs208688454 shows favorable sweat gland length properties with homozygous Angus alleles having significantly larger length (626 μm) when compared to homozygous Brahman alleles (609 μm).

## Conclusions

Throughout this study, considerable biological diversity was observed in the sweat gland characteristics being examined, suggesting good opportunities for selection and genetic improvement. Heritability was estimated to be 0.42 for sweat gland area, 0.28 for sweat gland depth, and 0.17 for sweat gland length. To our knowledge, this is the first report estimating heritability for sweat gland area and depth. These results suggest that a moderate to substantial amount of the phenotypic variation is due to genetic factors, providing a foundation for targeted breeding strategies to enhance these traits efficiently. This new knowledge can open up further research opportunities to explore the genetic basis of thermoregulation in cattle and other species. GWAS of sweat gland properties illustrated genes with functions related to the adaptation of these individuals regarding heat stress. Breed of origin GWAS results show favorable marker effects on sweat gland properties when inherited from either homozygous Brahman as well as homozygous Angus alleles. This might uncover specific genetic adaptations for heat tolerance that are not as prominent or necessary in the naturally heat-tolerant Brahman breed. This research is valuable for enhancing the resilience of cattle breeds less adapted to heat and for advancing our understanding of genetic adaptations to environmental stressors.

## Data Availability

Genomic data are available through the European Variation Archive (EVA), accession number PRJEB60100. Phenotypic data are available through Dryad, 10.5061/dryad.h70rxwdp5.

## Abbreviations

ADGRV1: Adhesion G protein-coupled receptor V1
AIREML: Average information restricted maximum likelihood
BOA: Breed of origin allele
BSM1: Bovine submaxillary mucin 1
CCDC168: Coiled-coil domain containing 168
ccRCC: Clear cell renal cell carcinoma
CL: Corpus luteum
FSIP2: Fibrous sheath interacting protein 2
GBLUP: Genomic best linear unbiased prediction
GWAS: Genome wide association study
LAMP-LD: Local ancestry in admixed populations
SNP: Single nucleotide polymorphism
STARD9: StAR related lipid transfer domain containing 9
QTL: Quantitative trait locus
WssGBLUP: Weighted single-step genomic best linear unbiased prediction
